# Supplementary material for: Comparative genomics provides new insights into the diversity, physiology, and sexuality of the only industrially exploited tremellomycete: Phaffia rhodozyma
Source: BMC Genomics. 2016 Nov 9;17:901. doi: 10.1186/s12864-016-3244-7 (PMC5103461; doi:10.1186/s12864-016-3244-7)
Supplement: Additional file 6: — List of orphan genes with links to PFAM (related to Additional file 1: Table S1). (ZIP 1428 kb) [file 12864_2016_3244_MOESM6_ESM.zip › PFAM_UNIQUE/G05688_P.html]

#                                                                            --- full sequence --- -------------- this domain -------------   hmm coord   ali coord   env coord
  
# target name        accession   tlen query name           accession   qlen   E-value  score  bias   #  of  c-Evalue  i-Evalue  score  bias  from    to  from    to  from    to  acc description of target
  
#------------------- ---------- ----- -------------------- ---------- ----- --------- ------ ----- --- --- --------- --------- ------ ----- ----- ----- ----- ----- ----- ----- ---- ---------------------
  
G05688\_P             -            570 CSF-1                PF05337.6    285       0.1   10.6   0.0   1   3      0.05     3e+02   -0.8   0.2   140   194    78   132    72   146 0.79 - Macrophage colony stimulating factor-1 (CSF-1)  
G05688\_P             -            570 CSF-1                PF05337.6    285       0.1   10.6   0.0   2   3   1.7e-05       0.1   10.6   0.0    92   131   250   292   226   304 0.85 - Macrophage colony stimulating factor-1 (CSF-1)  
G05688\_P             -            570 CSF-1                PF05337.6    285       0.1   10.6   0.0   3   3       0.3   1.8e+03   -3.4   1.8    96    96   425   425   338   483 0.57 - Macrophage colony stimulating factor-1 (CSF-1)  
G05688\_P             -            570 DUF3323              PF11796.3    215       7.7    4.4   7.5   1   2   0.00025      0.37    8.7   0.8    71   141    89   177    78   185 0.75 - Protein of unknown function N-terminus (DUF3323)  
G05688\_P             -            570 DUF3323              PF11796.3    215       7.7    4.4   7.5   2   2      0.39   5.9e+02   -1.7   0.9    70   105   302   334   296   349 0.62 - Protein of unknown function N-terminus (DUF3323)  
G05688\_P             -            570 FlgN                 PF05130.7    143       2.6    7.0   6.1   1   1    0.0075         5    6.0   6.1    50   131   107   177    85   181 0.79 - FlgN protein  
G05688\_P             -            570 HeLo                 PF14479.1    212       1.5    7.2   6.3   1   2   6.5e-05     0.097   11.1   0.8    55   137   135   219   118   295 0.86 - Prion-inhibition and propagation  
G05688\_P             -            570 HeLo                 PF14479.1    212       1.5    7.2   6.3   2   2       2.4   3.6e+03   -3.8   0.3   100   128   446   464   424   475 0.51 - Prion-inhibition and propagation  
G05688\_P             -            570 IncA                 PF04156.9    191     0.021   13.1   9.4   1   2     0.027       2.6    6.2   1.8    93   146   103   171    45   185 0.54 - IncA protein  
G05688\_P             -            570 IncA                 PF04156.9    191     0.021   13.1   9.4   2   2    0.0009     0.088   11.1   1.0    70   137   284   346   245   359 0.80 - IncA protein
